# STAT3 is required for Smo‐dependent signaling and mediates Smo‐targeted treatment resistance and tumorigenesis in Shh medulloblastoma

**DOI:** 10.1002/1878-0261.13097

**Published:** 2021-09-25

**Authors:** Liangping Yuan, Hongying Zhang, Jingbo Liu, Anshu Malhotra, Abhinav Dey, Bing Yu, Kishore Kumar Jella, Leon F. McSwain, Matthew J. Schniederjan, Tobey J. MacDonald

**Affiliations:** ^1^ Department of Pediatrics Emory University School of Medicine Atlanta GA USA; ^2^ Department of Pathology and Laboratory Medicine Children's Healthcare of Atlanta Emory University School of Medicine Atlanta GA USA; ^3^ Aflac Cancer & Blood Disorders Center Children's Healthcare of Atlanta Emory University School of Medicine Atlanta GA USA

**Keywords:** drug resistance, medulloblastoma, sonic hedgehog, STAT3, tumorigenesis

## Abstract

Sonic hedgehog (Shh)‐driven medulloblastoma (Shh MB) cells are dependent on constitutive Shh signaling, but targeted treatment of Shh MB has been ineffective due to drug resistance. The purpose of this study was to address the critical role of signal transducer and activator of transcription 3 (STAT3) in Shh signaling and drug resistance in Shh MB cells. Herein, we show that STAT3 is required for Smoothened (Smo)‐dependent Shh signaling and, in turn, is reciprocally regulated by Shh signaling, and demonstrate that STAT3 activity is critical for expression of HCK proto‐oncogene, Src family tyrosine kinase (Hck) in Shh MB. We also demonstrate that maintained STAT3 activity suppresses p21 expression and promotes colony formation of Shh MB cells, whereas dual treatment with inhibitors of both Smo and STAT3 results in marked synergistic killing and overcomes drug resistance *in vitro* of Smo antagonist‐resistant Shh MB cells. Finally, STAT3 inhibitor treatment significantly prevents *in vivo* tumor formation in genetically engineered Shh MB mice. Collectively, we show that STAT3 is necessary to maintain Shh signaling and thus is a potential therapeutic target to treat Shh MB and overcome anti‐Smo drug resistance.

## Graphical abstract

Medulloblastoma is the most common malignant brain tumor in children, but targeted treatment of sonic hedgehog (Shh) medulloblastoma has been ineffective due to drug resistance. In this study, we demonstrate that signal transducer and activator of transcription 3 is critical for Shh signaling and thus is a potential therapeutic target to treat Shh medulloblastoma and overcome drug resistance.

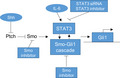

**Abbreviations**
Gli1: Gli family zinc finger 1
Hck: HCK proto‐oncogene, Src family tyrosine kinase
IC50: half‐maximal inhibitory concentration
IFN‐α: interferon‐α
IL‐6: interleukin 6
MB: medulloblastoma
MTT: 3‐(4,5‐dimethylthiazol‐2‐yl)‐2,5‐diphenyltetrazolium bromide
p21: cyclin‐dependent kinase inhibitor 1A
p‐STAT3: phosphorylated STAT3
Ptch1: Patched 1
SAG: Smoothened Agonist
Shh: sonic hedgehog
shRNA: short hairpin RNA
siRNA: small interference RNA
Smo: Smoothened
Sox2: SRY‐box transcription factor 2
STAT3: signal transducer and activator of transcription 3

## 1 Introduction

Medulloblastoma (MB) is the most common malignant brain tumor in children and consists of four subgroups [WNT, sonic hedgehog (Shh), Group 3, and Group 4] [1, 2, 3]. Shh MB displaying one of the following characteristics: large cell anaplastic histology, *MYCN* amplification, *TP53* mutation, or metastasis, constitutes an extremely high‐risk group with poor prognosis [4]. Shh MB is dependent on activation of the Shh signaling pathway for tumor initiation, survival, and growth [5, 6]. Normally, Shh signaling is initiated by Shh binding to Patched 1 (Ptch1), relieving its repression of Smoothened (Smo), resulting in Gli transport to the nucleus and transcriptional activity [5, 7]. Somatic mutations in Shh MB that include *Ptch1*, *Smo*, and *SUFU*, a negative regulator that functions by maintaining Gli in the cytoplasm until Smo activation, and amplification of Shh effectors, *MYCN*, *Gli1,* and *Gli2*, result in constitutive Shh signaling [4, 8, 9, 10]. These intrinsic and acquired mutations, and amplifications can contribute to Smo‐targeted therapeutic resistance [11, 12, 13]. Smo‐targeted Shh MB was shown to develop early treatment resistance due to specific cell lineages with persistent Shh pathway activation [14]. Alternative targeting of aberrant Shh signaling thus has important implications for the development of novel therapeutics against Shh MB [15, 16].

Amplified oncogenes, such as *ADAM29*, *MYCN*, *BOC*, and *CDK6*, are associated with Shh MB [17], while specific growth factors (e.g., IGF, PDGF) have been associated with Shh signaling [18, 19]. Our previous studies demonstrated that PDGFRA, CXCL12, and CXCR4 are highly expressed in Shh MB [20] and are significantly associated with MB metastasis and clinical outcomes [21, 22, 23]. Signal transducer and activator of transcription 3 (STAT3) have critical regulatory interactions with most of these Shh‐associated oncogenes and growth factor‐mediated signaling pathways, and plays an essential role in cancer stem cell maintenance, tumorigenesis, and cancer progression [24, 25]. Evidence shows that STAT3 is highly activated in Shh‐dependent carcinomas. Expression level of p‐STAT3 (phosphorylation at Tyr705) directly correlates with ligand‐dependent activation of Shh signaling in pulmonary adenocarcinoma [26], and removal of STAT3 from mouse epidermis dramatically reduced SmoM2‐mediated basal cell carcinoma development [27]. Constitutive activation of STAT3 has been reported in MB, and multiple studies have shown that targeting STAT3, directly and indirectly, can inhibit MB cell survival [28, 29, 30]. Importantly, tumor‐associated astrocyte secretion of Shh drives expression of the stem cell marker nestin in Ptch1‐deficient granule neuron precursors and is indispensable for Shh MB tumorigenesis in a Smo‐dependent, but Ptch1‐ and Gli1‐independent manner [31]. However, the key mediators of this novel Shh pathway remain unclear. Based on STAT3's role in stem cell maintenance and associations with MB, we tested whether STAT3 plays a critical role in Shh‐induced Smo‐dependent signaling and Shh MB tumorigenesis.

## 2 Materials and methods

### 2.1 Cell culture and reagents

Daoy and D556 human medulloblastoma cells were obtained from ATCC (Manassas, VA, USA) and cultured in EMEM with 10% FBS. UW228 and ONS‐76 human medulloblastoma cells were kindly provided by C. Eberhart (Johns Hopkins University, Baltimore, MD) and were cultured in DMEM/F‐12 medium with 10% FBS. SMB21 and SMB21‐Gli2 ΔN cell lines were gifts from R. Segal (Harvard University) and were cultured in DMEM/F12 medium with B27.

Preparation of primary mouse Shh MB cells: cerebellum of NeuroD2‐SmoA1 (SmoA1) spontaneous MB mice with Smoothened overexpression was isolated into Dulbecco's PBS (DPBS) and digested with Papain and DNase at 37 °C for 15 min. Tissue was dissociated with a Pasteur pipette in ovomucoid and DNase solution prior to 65% Percoll fractionation. Cells were washed in DBPS + BSA prior to resuspension in Neurobasal media supplemented with B27, sodium pyruvate, and glutamine. 1.5 × 10^6^ or 3 × 10^6^ cells were plated in 1.5 mL of media per well onto Matrigel‐coated 6‐well plates and allowed to incubate for 24 h prior to treatment.

Smoothened inhibitor Sant‐1(cat# 559303), STAT3 inhibitor WP1066 (cat# 573097), and human interferon‐α (cat# SRP4596) were purchased from MilliporeSigma (St. Louis, MO, USA). Smoothened inhibitor LDE225 (cat# S2151) and STAT3 inhibitor NSC74859 (also named as S3I‐201, cat# S1155) were purchased from Selleckchem.com (Houston, TX, USA). Interleukin 6 (IL‐6) was purchased from R&D Systems (Minneapolis, MN, USA).

### 2.2 Western blot

Anti‐Gli1 (cat# 2553), anticleaved caspase‐3 (5A1E, cat# 9664), anti‐STAT3 (cat# 9139), and anti‐p‐STAT3 (Tyr705) (cat# 4113) antibodies were purchased from Cell Signaling Technology (Danvers, MA, USA). Anti‐p21 (F‐5, sc‐6246) and anti‐Sox2 (E‐4, sc‐365823) antibodies were purchased from Santa Cruz Biotechnology (Dallas, TX, USA). Western blot of whole‐cell lysates harvested in lysis buffer was performed with primary antibodies, and then, goat anti‐mouse or rabbit horseradish peroxidase secondary antibodies were used and the immunoreactive bands were detected by using SuperSignal West Pico Chemiluminescent substrate, or SuperSignal West Dura Extended Duration substrate (Thermo Fisher Scientific, Waltham, MA, USA). All experiments for western blot were performed at least three times except where indicated in the figure legends. Western blot protein bands were quantified by using imagej, the ratio of each protein band relative to the lane's loading control was calculated, and the final relative values are the ratio of net protein band to net loading control. The ratio for each control cell was normalized as 1, then calculated the relative fold change for each lane relative to the control in each cell line.

### 2.3 Real‐time RT‐PCR

Total RNA was prepared from the human MB cell lines or mice cerebellum tissue. Random‐primed single‐stranded cDNA was made from total RNA by using the Superscript III kit (Cat# 18080‐051; Thermo Fisher Scientific). The following oligonucleotides as primers were used for real‐time PCR: Glyceraldehyde‐3‐phosphate dehydrogenase (*GAPDH*), 5′‐ACAGTCCATGCCATCACTGCC‐3′ (forward), 5′‐GCCTGCTTCACCACCTTCTTG‐3′ (reverse); human *Gli1*, 5′‐ATGGAGAGAGCCCGCTTCTTT‐3′ (forward), 5′‐TTATGGAGCAGCCAGAGAGACCAG‐3′ (reverse); human *STAT3*, 5′‐GGAGGAGTTGCAGCAAAAAG‐3′ (forward), 5′‐TGTGTTTGTGCCCAGAATGT‐3′ (reverse); human *Hck*, 5′‐CGGATCCCACATCCACCATCA‐3′ (forward), 5′‐ACCACGATGATGTCCTCAGAGC‐3′ (reverse); murine *Ptc*, 5′‐GGCAAGTTTTTGGTTGTGGGTC‐3′ (forward), 5′‐CCTCTTCTCCTATCTTCTGACGGG‐3′ (reverse). Data analysis was performed according to the absolute standard curve method. Data are presented in relation to the respective housekeeping gene and normalized the fold change of control cells to 1 (100%), then calculated the relative fold changes.

### 2.4 siRNA transfection

Human STAT3 ON‐TARGETplus SMARTpool siRNA (cat# L‐003544‐00‐0005), and negative control nontargeting siRNA (D‐001810‐01‐05) were purchased from GE Healthcare Dharmacon, Inc (Lafayette, CO, USA). Each SMARTpool is a mixture of four siRNA sequences with advantages in both efficacy and specificity. For siRNA transfections, 1.5 × 10^5^ cells were seeded in each well of a six‐well plate and grown to 50–70% confluency prior to transfection. Cells were transfected with siRNA using Lipofectamine 2000 (Thermo Fisher Scientific) for 48–72 h according to the manufacturer's instruction, and then, cells were split, or harvested with or without treatment for experiments.

### 2.5 MTT assay

Daoy cells were cultured in 96‐well plates at a density of 1 × 10^4^ cells in medium containing 3% FBS and incubated for 24 h. After treatment, 10 µL MTT solution (5 mg·mL^−1^) was added into each well and incubated for 3 h, then the medium containing MTT was removed, and then 100 µL isopropanol with 0.04 N HCL was added into each well, and shaken gently for 5 min. Absorbance was measured at 570 nm.

### 2.6 Cell proliferation assay (WST‐1 assay)

Cell proliferation reagent WST‐1 was purchased from Roche (Indianapolis, IN, USA; cat# 05 015 944 001). Cells were seeded into 96‐well plates, with 100 µL volume in each well, grown for 24–72 h (for attached cells, depending on experiments), then given different treatment as described following the cell growth. For suspension cells, cells were added into 96‐well plates, then given different treatment as described. Same volume medium without cells in each well was used for background control. Ten microliter WST‐1 solution was added into each well and incubated for 2–4 h at 37 °C; then, the OD value was read at 450 nm. OD value for each well was calculated by each OD value subtracting the average OD value of the background control group.

### 2.7 Gli1 reporter assay

Dual‐luciferase reporter assay system kit was purchased from Promega (cat# E1910, Madison, WI, USA). Shh Light II cells (a gift from M. Resh, Memorial Sloan‐Kettering Cancer Center) were used to examine Gli1 reporter activity; each treatment was set in triplicate. Cells were pretreated with DMSO vehicle, Smo inhibitor Sant‐1, or STAT3 inhibitor NSC74859 for 1 h, then given different treatment as described. After treatment, cells were washed with PBS and 1× PLB lysis buffer added into each well. Harvested cell lysates were used to measure firefly luciferase/Renilla luciferase and the ratio and fold changes were calculated.

### 2.8 Stable cell lines

#### 2.8.1 Constitutively activated STAT3 cells

Plasmids p‐IRES‐EGFP (control vector) and p‐IRES‐S3c (constitutively activated STAT3) were gifts from B. Barton (New Jersey Medical School, Rutgers University). p‐IRES‐EGFP or p‐IRES‐S3c was transfected into Daoy cells by Lipofectamine 2000, respectively. Twenty‐four hours after transfection, G418 was added to treat cells of each group until the Daoy cells without transfection of plasmids (control cells) were completely killed (about 10 days). Finally, the transfected cells were sorted by flow cytometry to select cells with EGFP. Frozen the stable cells for further experiments.

#### 2.8.2 STAT3 knockdown cells

STAT3 shRNA plasmid (h) (#sc‐29493) and control shRNA(#sc‐108080) were purchased from Santa Cruz Biotechnology. Puromycin was used to select Daoy or D556 cells transfected with human STAT3 shRNA or control shRNA. After removing the medium with dead cells under puromycin selection, single colonies were picked up from the culture dishes and then put into single wells in a new 96‐well plate to grow. The cells were kept growing in the same medium with puromycin until enough cells were isolated to confirm the resulting expression of STAT3 by western blot.

### 2.9 Colony formation assay

Stable Daoy cells with p‐IRES‐EGFP or p‐IRES‐S3c were seeded into 10‐cm cell culture dishes, 200 cells/dish, and three dishes for each treatment. Let cells grow in culture medium with 10% FBS 24 h and then treat with vehicle or Sant‐1 for 24 h. After treatment, medium was changed and let cells grow for another 7 days. Cells were then fixed and stained, and the colonies were counted.

### 2.10 Flow cytometry analysis

For flow cytometry analysis, Daoy or ONS‐76 cells were treated with vehicle (DMSO), or different doses of NSC74859 or Sant‐1 as indicated, alone and in combination, for 48 h to determine drug‐induced apoptosis. For cell apoptosis profiling, treated cells were stained with Alexa Fluor 488 Annexin V/Dead Cell Apoptosis and PI (Thermo Fisher Scientific). Results were acquired with a BD FACSymphony™ A5 Cell Analyzers (BD Biosciences, Franklin Lakes, NJ, USA) and analyzed with flowjo 10 (Tree Star, Inc., Ashland, OR, USA).

### 2.11 Immunohistochemistry

Tissues were fixed in 10% neutral‐buffered formalin, processed, and embedded in paraffin. Five‐micrometer‐thick sections were deparaffinized in xylene and rehydrated to water prior to microwave antigen retrieval in Tris/HCl/EDTA pH 9.0 buffer (Dako Cytomation, Glostrup, Denmark) and PBS washing. After neutralization of the endogenous peroxidase with 3% H_2_O_2_ for 10 min, the sections were incubated with protein blocking buffer for 10 min before undergoing incubation with the primary antibody. Anti‐Patched (Santa Cruz Biotechnology) staining was developed using DAB (Vector Laboratories, Burlingame, CA, USA) followed by Hematoxylin counterstaining (MilliporeSigma). H&E staining was performed on 5‐μm paraffin sections according to common methods.

### 2.12 *In* *vivo* studies

SmoA1 mice were purchased from the Jackson Laboratory (Bar Harbor, ME, USA). Math1‐Cre‐ER‐*Ptc* ^flox/flox^ mice were generated as described [32] and used for *in vivo* experiments after obtaining IACUC approval. The mice were maintained in Emory University animal facilities approved by the American Association for Accreditation of Laboratory Animal Care. The protocol number from the authorities for animal is PROTO 201700138. The Division of Animal Resources (DAR) provides the daily maintenance or routine animal care, and the Veterinary Services Unit at DAR provides the entire animal health care program, which includes vendor surveillance, quarantine and isolation, preventive medicine, daily observation, treatment and intervention for injury or illness, and health evaluations of sentinel animals. The pregnant female mice were administered tamoxifen (T‐5648; MilliporeSigma) by oral gavage using 24‐G gavaging needles (Fine Science Tools, Foster City, CA, USA) after E17.0 to induce Shh MB formation. Tamoxifen was prepared as a 20 mg·mL^−1^ solution in corn oil (MilliporeSigma), and the tamoxifen dose was 4 mg/200 μL for treatment of pregnant females. After the pups were born for 1 week, they were given NSC74859 at dose of 50 mg·kg^−1^ I.P., every other day for 2 weeks prior to tumor formation by histological examination. NSC 74859 stock solution (70 mg·mL^−1^, in DMSO) was made first and then prepared as a 10 mg·mL^−1^ solution by dilution at 1 : 7 in PBS for the I.P. administration in a colloidal suspension solution, 5 mL·kg^−1^ for each pup. The mice were observed weekly until death or the end of the experiment at 40 weeks.

### 2.13 Statistical analysis

Isobolographic analysis was used to calculate drug synergy, whereby the Combination Index (CI) is used to determine the degree of drug interaction:

CI=Ca/IC50a+Cb/IC50b,

where IC50a is the IC50 value of drug a; IC50b is the IC50 value of drug b; Ca is the concentration of drug a in the combination experiment; and Cb is the concentration of drug b in the combination experiment. If CI ≤ 1, there is synergy effect of the two drugs; CI = 1 there is addition effect of the two drugs; CI ≥ 1 there is antagonistic effect of the two drugs. For flow cytometry data, graphpad prism 8.0 (GraphPad Software Inc., San Diego, CA, USA) software was used to calculate statistical significance. For comparisons across groups, we used one‐way analysis of variance followed by Dunnett's *post hoc* test; For other *in vitro* experiments, Student's *t*‐test was used to perform statistical analysis; Fisher's exact test was used for *in vivo* study.

## 3 Results

### 3.1 STAT3 activation and expression in Shh MB cells are Smo‐dependent

**Fig. 1:**
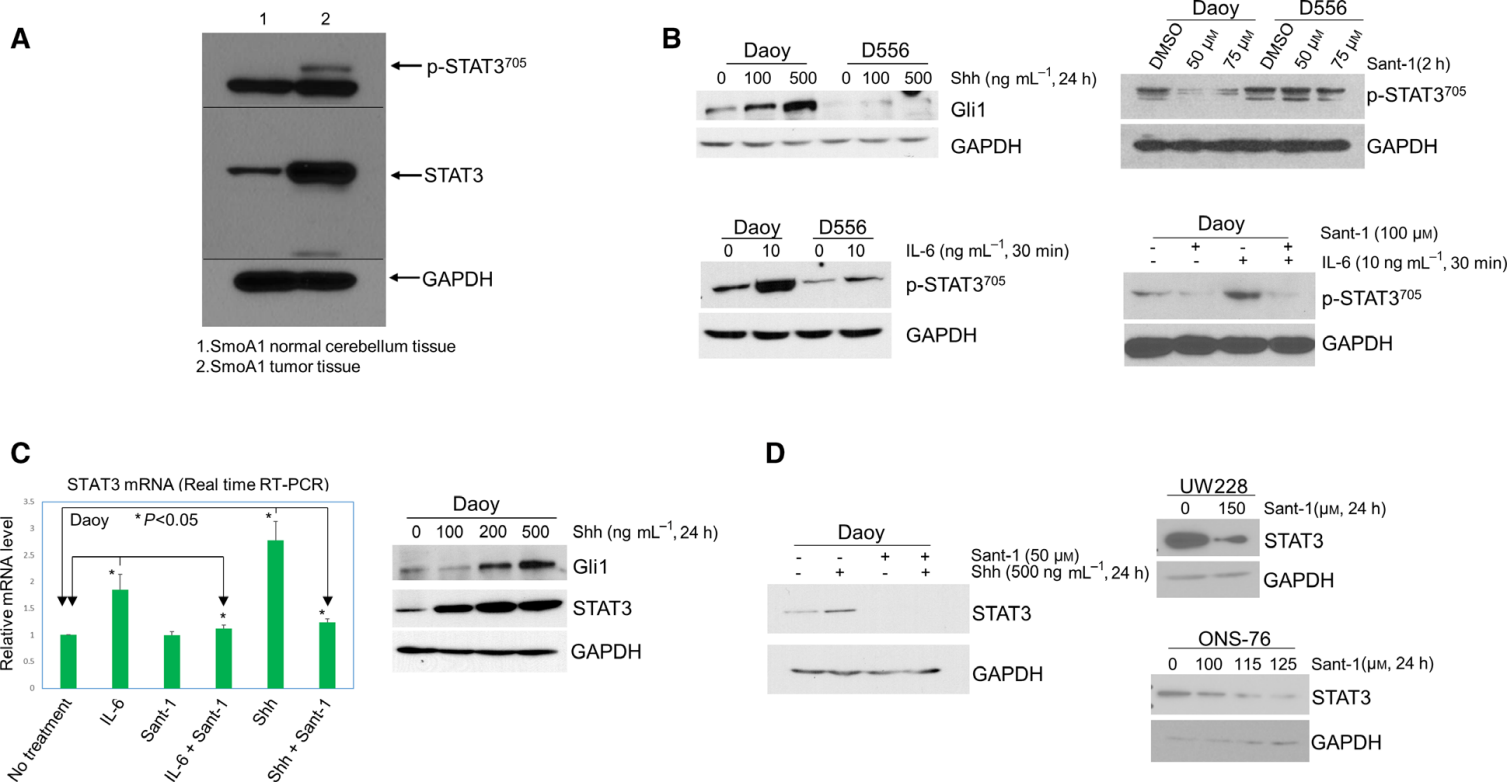
STAT3 activation and expression in Shh MB cells are Smo‐dependent. (A) western blot of p‐STAT3^705^ and total STAT3 in normal cerebellum tissue and tumor tissue from SmoA1 mice. (B) Upper left panel: Gli1 in Daoy and D556 cells after Shh treatment; upper right panel: western blot showing the effect of Sant‐1 treatment on p‐STAT3^705^ in Daoy and D556 cells; bottom left panel: IL‐6‐induced expression of p‐STAT3^705^ in Daoy and D556 cells; bottom right panel: Sant‐1 blocked IL‐6‐induced p‐STAT3^705^ in Daoy cells. (C) Left panel: quantitative real‐time RT‐PCR for expression of *STAT3* mRNA. Daoy cells were treated with IL‐6 (20 ng·mL^−1^) or Shh (500 ng·mL^−1^) for 6 h; then, total RNA was extracted from the cells for PCR, at least in triplicate for each sample for each PCR. The relative amount of *STAT3* mRNA was increased 1.85‐fold by IL‐6, or 2.78‐fold by Shh, compared with the untreated cells, *P* < 0.05, respectively. Sant‐1(75 µm) significantly blocked IL‐6 or Shh‐induced *STAT3* mRNA. Data are presented as mean ± SEM (*n* = 3); *t*‐tests were used to determine the significance; right panel: western blot of Gli1 and STAT3 increased following Shh treatment at different doses. (D) Western blot of Shh induced increase of STAT3 protein blocked by Sant‐1 in Daoy cells (left panel); Sant‐1 treatment inhibited expression of STAT3 protein in UW228 and ONS‐76 cells (right panels). All western blot results shown in this figure are representative of at least three independent experiments.

Because IL‐6 has been shown to activate STAT3 in Shh MB cells [33], and p‐STAT3 levels correlate with Shh signaling in other cancers [26, 34], to determine whether Shh signaling is associated with STAT3 in MB, we examined expression of p‐STAT3 and STAT3 in normal cerebellum and tumor tissue derived from SmoA1 mice, and each normal cerebellum and tumor tissue tested was from same adult mouse, which express constitutively activated Smo in cerebellar granule neuron precursors resulting in Shh MB [35]. We observed high expression of p‐STAT3 and total STAT3 in murine Shh MB compared with normal cerebellum (Fig. 1A), suggesting the association between STAT3 and Shh MB. To test the effect of STAT3 inhibition in our Shh MB murine model, we treated the primary mouse Shh MB cells derived from SmoA1 mice with the STAT3 inhibitor NSC74859 and observed decreased p‐STAT3 and total STAT3 in murine Shh MB cells compared with control treatment (Fig. S1A), confirming the SmoA1 MB model is appropriately responsive to the drug. To further determine whether STAT3 activity correlates with Shh signaling, we then examined expression of the Shh pathway effector Gli1 in Shh MB and non‐Shh MB cells. Shh treatment (24 h) induced Gli1 expression in human Shh MB cells (Daoy) in a dose‐dependent manner, but had little effect on human D556 non‐Shh MB cells (Fig. 1B, upper panel, left). Furthermore, Sant‐1 treatment (2 h) induced marked reduction of p‐STAT3 in Shh MB cells relative to control treatment, but had only minimal effect on expression of p‐STAT3 in D556 non‐Shh MB cells (Fig. 1B, upper panel, right). Similarly, treatment with IL‐6, the primary activator of STAT3, induced p‐STAT3^705^ at much higher levels in Shh MB cells relative to non‐Shh MB cells (Fig. 1B, bottom panel, left). Accordingly, IL‐6‐mediated induction of p‐STAT3 was abolished by pretreatment with the Smo inhibitor, Sant‐1 (Fig. 1B, bottom panel, right); however, Sant‐1 had little inhibitory effect on IL‐6‐mediated induction of p‐STAT3 in D556 non‐Shh MB cells (Fig. S1B). Additionally, interferon‐α (IFN‐α)‐induced p‐STAT3^705^ was also blocked by Sant‐1 treatment in two separate human Shh MB cells (Fig. S1C). To further confirm that STAT3 activity is Smo‐dependent, we used the clinical Smo inhibitor LDE225 to treat Shh MB cells and examine p‐STAT3 and STAT3 to see whether LDE225 has a similar effect on inhibiting STAT3 and to confirm our results are not off‐target effects unique to Sant‐1. LDE225 treatment, similar to Sant‐1, inhibited STAT3 activation and expression in Shh MB cells (Fig. S1D). We then addressed the effect of Shh signaling on STAT3 expression. As shown in Fig. 1C, Shh treatment induced *STAT3* mRNA expression by 2.78‐fold, relative to control cells (*P* < 0.05), and this effect was greater than IL‐6 treatment, which increased *STAT3* mRNA level by 1.85‐fold compared with control (*P* < 0.05). Similar to IL‐6‐mediated p‐STAT3 induction, IL‐6‐ or Shh‐mediated *STAT3* mRNA induction was blocked by Sant‐1 pretreatment (Fig. 1C, left panel). STAT3 protein expression following Shh treatment (24 h) similarly increased in a dose‐dependent manner and directly correlated with Gli1 protein levels after Shh treatment (Fig. 1C, right panel). Furthermore, STAT3 protein expression was markedly inhibited by Sant‐1 in three separate Shh MB cells (Fig. 1D). These data indicate that STAT3 activation and expression in Shh MB are Smo‐dependent.

### 3.2 Smo‐mediated Shh signaling in Shh MB cells is reciprocally dependent on STAT3

**Fig. 2:**
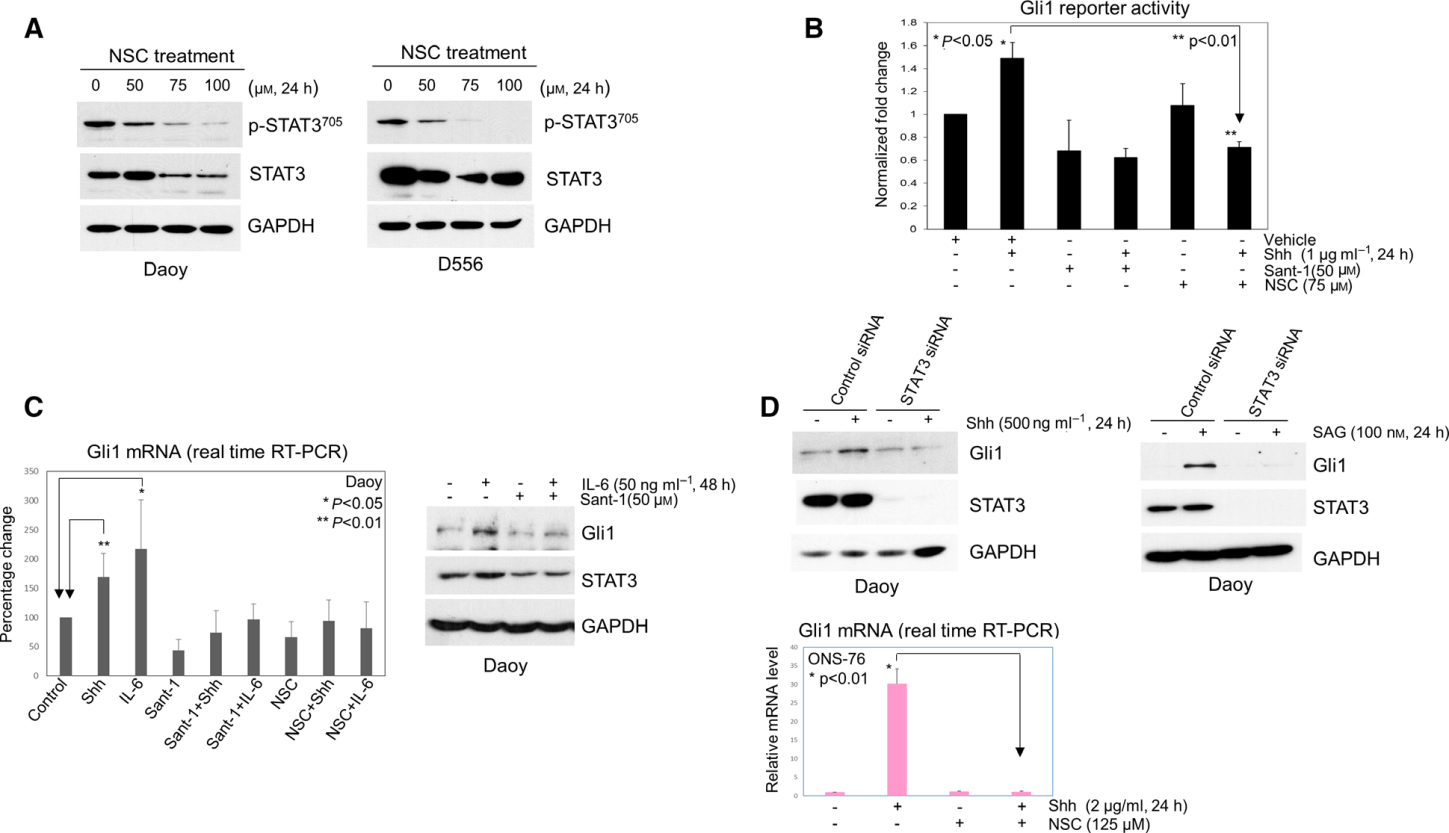
Smo‐mediated Shh signaling in Shh MB cells is reciprocally dependent on STAT3. (A) Western blot showing NSC74859 (NSC) treatment abolishes expression of p‐STAT3 and concomitantly reduces total STAT3 in Daoy and D556 cells. (B) Gli1 reporter assays were performed to determine relative Gli1 reporter activity in Shh Light II cells; each treatment was set in triplicate. The cells were treated with vehicle or Shh alone, or Shh combined with Sant‐1 or NSC74859 for 24 h. Shh alone significantly increased Gli1 reporter activity (1.5‐fold, *P* < 0.05), but NSC74859 treatment significantly decreased Shh‐induced Gli1 reporter activity, only 0.7‐fold, compared with the untreated cells, significantly lower than in the cells with Shh treatment alone (*P* < 0.01). Data are presented as mean ± SD; *t*‐tests were used to determine the significance. Results shown are representative of at least three independent experiments. (C) Left panel: Expression of *Gli1* mRNA was examined by RT‐PCR in Daoy cells. Shh treatment (500 ng·mL^−1^, 6 h) alone significantly increased relative *Gli1*mRNA expression, 1.7‐fold higher than untreated group (*P* < 0.01). IL‐6 treatment (50 ng·mL^−1^, 6 h) alone increased *Gli1* mRNA 2.2‐fold, significantly higher than control treatment (*P* < 0.05), and Shh‐induced or IL‐6‐induced *Gli1* mRNA was completely blocked by Sant‐1(100 µm) or NSC74859 (100 µm). Data are presented as mean ± SEM (*n* = 6); *t*‐tests were used to determine the significance; right panel: western blot showing IL‐6 increased expression of Gli1 and STAT3 protein in Daoy cells and blocked by Sant‐1 treatment. (D) Upper panels: Daoy cells were first transfected with control siRNA or STAT3 siRNA, then treated with vehicle, or Shh or SAG (activator of Smo), Gli1 and STAT3 protein were examined by western blot; bottom panel: showing Shh treatment increased *Gli1* mRNA 30‐fold in ONS‐76 cells, but NSC74859 completely blocked Shh‐induced *Gli1* mRNA (*P* < 0.01). Data are presented as mean ± SD; *t*‐test was used to determine the significance. Results shown are representative of at least three independent experiments. All western blot results shown in this figure are representative of at least three independent experiments.

Gli1 is a marker of Shh pathway activation that promotes Shh MB survival and tumorigenesis [5, 36, 37, 38]. Gli1 target genes include *Ptch1*, *Gli*, and *cyclin D* [5, 38, 39]. To address whether STAT3 plays a role in Gli1 regulation, we examined the effect of STAT3 inhibition on Shh‐induced Gli1 reporter activity using Shh Light II cells expressing a Gli1‐responsive reporter [40]. First, we examined whether the STAT3 inhibitor NSC74859 sufficiently blocks p‐STAT3 in human MB cells. As shown in Fig. 2A, NSC74859 treatment of Shh and non‐Shh human MB cells effectively inhibits expression of p‐STAT3^705^ in a dose‐dependent manner. In addition, NSC74859 treatment also prevents IL‐6‐induced p‐STAT3 in Shh MB cells (Fig. S2A). We then tested Gli1 reporter activity, with vehicle control treated Shh Light II cells Gli1 reporter luciferase activity normalized to 1, and as shown in Fig. 2B, Shh treatment (1 µg·mL^−1^, 24 h) increased Gli1 activity to 1.5‐fold higher compared with untreated cells (*P* < 0.05), while Sant‐1 pretreatment completely blocked Shh‐induced Gli1 activity. Similar to Sant‐1 treatment, STAT3 inhibitor (NSC74859) pretreatment completely blocked Shh‐induced Gli1 activity (0.7‐fold of untreated control cells), and Shh‐induced Gli1 activity after NSC74859 pretreatment was significantly lower than in the cells with Shh treatment alone (*P* < 0.01). To further elucidate the role of STAT3 in Shh signaling, we treated Shh MB cells with the STAT3 activator IL‐6 and examined *Gli1* mRNA expression. As shown in Fig. 2C, Shh treatment increased *Gli1* mRNA expression 1.7‐fold higher compared with control cells (*P* < 0.01). Moreover, IL‐6 treatment alone increased *Gli1* mRNA expression level by 2.2‐fold relative to control cells (*P* < 0.05), and both Shh‐ and IL‐6‐mediated *Gli1* mRNA expression was completely blocked by either Sant‐1 or NSC74859 pretreatment. As shown in Fig. 2C (right panel), IL‐6 treatment was confirmed to increase the corresponding protein expression of Gli1 and STAT3. However, Sant‐1 pretreatment blocked IL‐6‐induced Gli1 and STAT3 expression, indicating that the IL‐6 effects on Gli1 and STAT3 are reciprocally dependent on Smo. To confirm the essential role of STAT3 in Shh signaling, we used STAT3 siRNA to knockdown STAT3 and then examined Gli1 expression. In cells with control siRNA, treatment with either Shh or SAG (an activator of Smo) induced marked expression of Gli1; however, Shh‐ and SAG‐induced Gli1 expression was abolished in STAT3 knockdown cells (Fig. 2D, upper panels). Identical results were observed in another Shh MB cell line, ONS‐76, following STAT3 inhibitor treatment (Fig. 2D, bottom panel).

### 3.3 STAT3 activation is required for Shh MB cell proliferation and survival

**Fig. 3:**
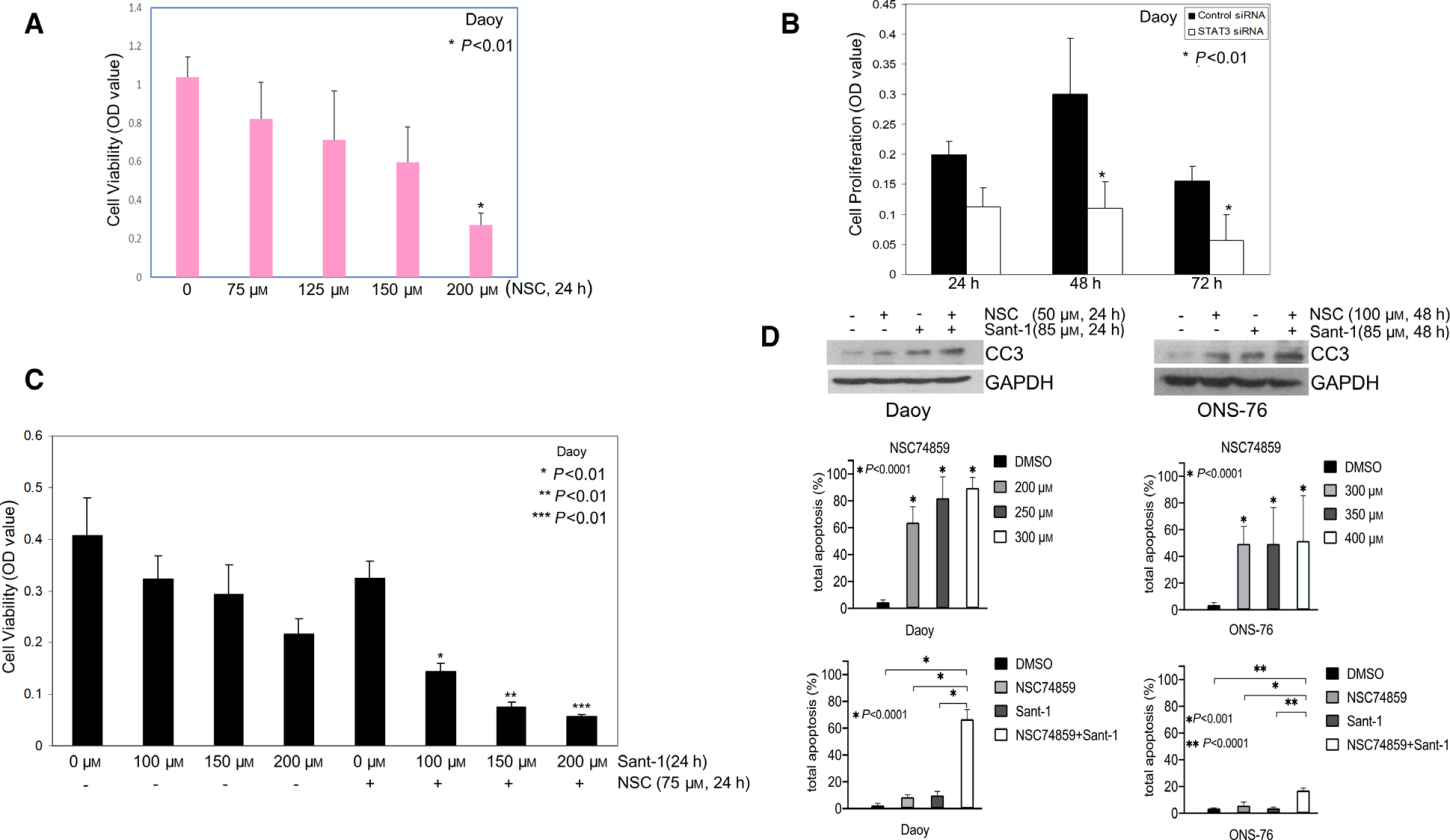
STAT3 activation is required for Shh MB cell proliferation and survival. (A) Daoy cells were seeded into 96‐well plates, each well contained 1 × 10^4^ cells in culture medium with 3% sera. The cells were treated with vehicle or NSC74859; then, MTT assay was performed to examine cell viability. NSC74859 treatment significantly decreased cell viability in a dose‐dependent manner. (B) Daoy cells were transfected with control siRNA or STAT3 siRNA for 72 h then split transfected cells into 96‐well plates to grow the cells in culture medium with 1% sera. WST‐1 assays were performed showing decreased cell proliferation with STAT3 knockdown. (C) MTT assay showing Sant‐1 treatment alone decreased cell viability, and combination of NSC74859 and Sant‐1 treatment resulted in a significant synergistic inhibition of cell viability, compared with corresponding Sant‐1 treatment doses alone (*P* < 0.01, respectively). (A–C) Data are presented as mean ± SD; *t*‐tests were used to determine the significance. Results shown are representative of at least three independent experiments. (D) Upper panels: western blot of cleaved caspase‐3 (CC3) showing NSC74859 or Sant‐1 treatment alone increases level of CC3 in Daoy and ONS‐76 cells, and combined treatment markedly enhances level of CC3. Results shown are representative of at least two independent experiments; middle and bottom panels: relative cell apoptosis was determined by flow cytometry analysis through dual staining of Annexin V/Dead Cell Apoptosis and PI. NSC74859 treatment (48 h) significantly induced apoptosis in both Daoy and ONS‐76 cells, and combination of low‐dose NSC74859 (100 µm) and Sant‐1 (85 µm) (48 h) synergistically promoted apoptosis in Daoy and enhanced apoptosis in ONS‐76 relative to each treatment alone. Data are presented as mean ± SD (*n* = 3). Statistics: One‐way analysis of variance followed by Dunnett's *post hoc* test.

To determine the impact of STAT3 on Shh MB cell growth, we treated cells with vehicle control or the STAT3 inhibitor NSC74859 for 24 h and then analyzed cell proliferation. As shown in Fig. 3, Shh MB cell (Daoy) growth significantly decreased in a dose‐dependent manner after NSC74859 treatment, with 200 µm NSC74859 treatment resulting in decreased cell growth by 74% compared with control cells (*P* < 0.01); similar results were observed in another two Shh MB cells (ONS‐76 and UW228) (Fig. S2B). To confirm the critical role of STAT3, Daoy Shh MB cells with STAT3 siRNA knockdown were similarly analyzed. As shown in Fig. 3B, 24 h after cells were seeded, the relative proliferation of the cells transfected with control siRNA is almost twofold higher than cells with STAT3 knockdown. The relative cell proliferation further drops by 63–64% (at 48 and 72 h, respectively) in STAT3 knockdown cells compared with control cells (*P* < 0.01). Because Sant‐1 treatment significantly inhibits Shh‐dependent cancer cell growth [41, 42], we tested whether combining treatment with NSC74859 enhances the anti‐tumor effects of Sant‐1 on Shh MB cells. Cells were treated with DMSO vehicle control, Sant‐1 or NSC74859 alone, or NSC74859 in combination with Sant‐1 for 24 h. As shown in Fig. 3C, Sant‐1 treatment significantly decreased cell growth, compared with the DMSO‐treated control cells, while low‐dose NSC74859 treatment (75 µm) only slightly decreased cell growth. However, the combination treatment of low‐dose NSC74859 with escalating doses of Sant‐1 resulted in a significant dose‐dependent and synergistic effect on reducing cell viability, compared with corresponding doses of Sant‐1 treatment alone (*P* < 0.01).

To assess the effect of combined treatment on survival, we measured induction of apoptosis by western blot for cleaved caspase‐3 and by flow cytometry for analysis of dual staining of Annexin V/dead cell apoptosis and PI. NSC74859 or Sant‐1 treatment alone increased cleaved caspase‐3 levels in Daoy and ONS‐76 cells relative to DMSO control treatment; however, combination treatment with NSC74859 and Sant‐1 markedly increased cleaved caspase‐3, compared with each treatment alone (Fig. 3D, upper panels). Consistent with these results, flow cytometry showed that NSC74859 treatment significantly induced apoptosis in both Daoy and ONS‐76 cells, and combination treatment with low‐dose NSC74859 (100 µm) and Sant‐1 (85 µm) resulted in significantly more apoptosis than NSC74859 or Sant‐1 treatment alone (Fig. 3D, lower panels).

### 3.4 STAT3 mediates resistance to Smo‐targeted treatment *in vitro*

**Fig. 4:**
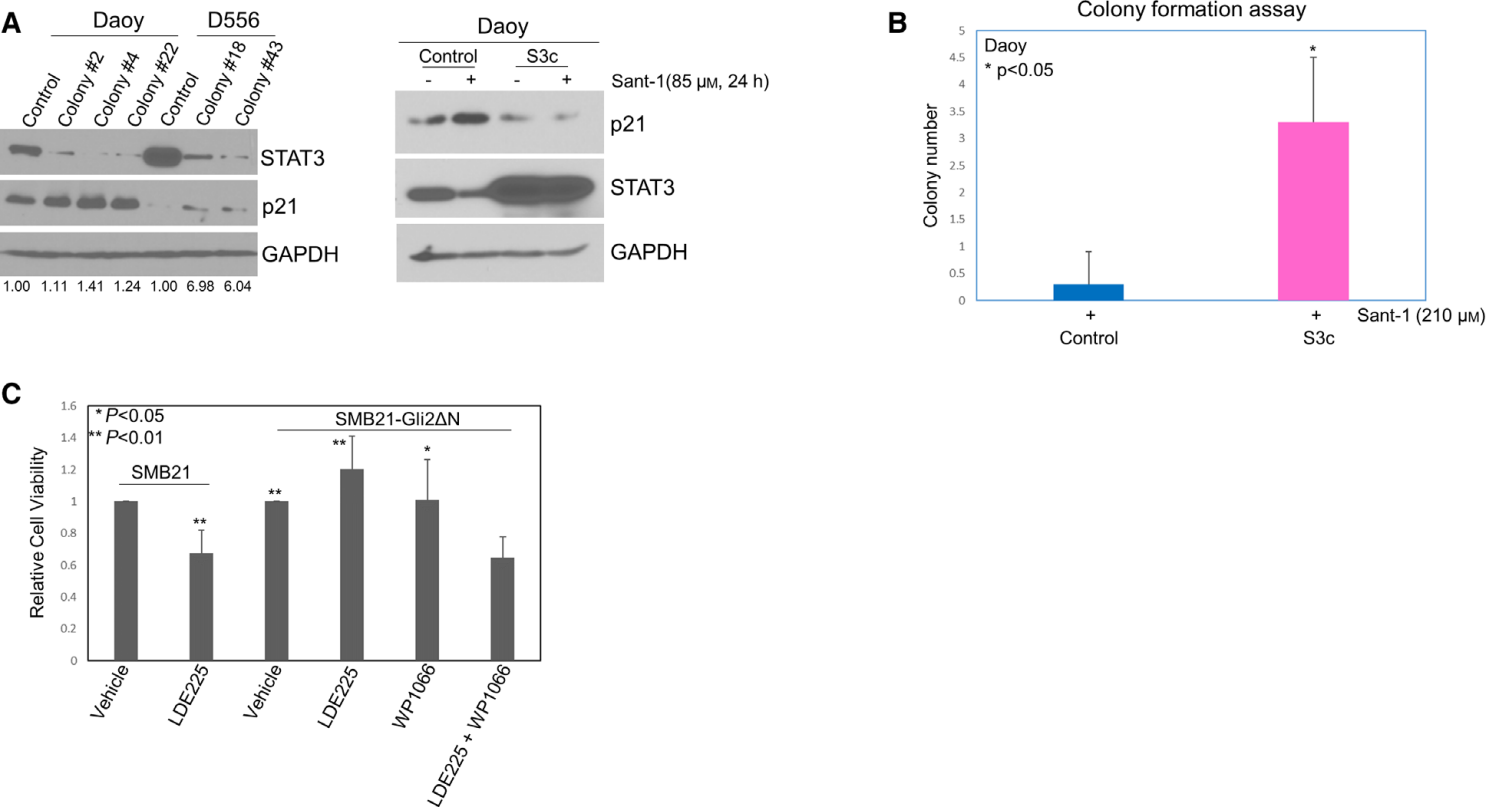
STAT3 mediates resistance to Smo‐targeted treatment *in vitro*. (A) Left panel: Cells from different single colonies of Daoy or D556 cells transfected with control shRNA or STAT3 shRNA were used to examine the level of p21 protein by western blot. p21 level was elevated in the colonies with knockdown of STAT3, compared with the control cells (the number on the bottom of the panel showing the fold changes relative to each control); right panel: western blot showing Sant‐1 treatment markedly increased p21 expression in stable Daoy cells with control vector, but Sant‐1‐induced p21 expression was inhibited in the cells with constitutively activated STAT3 vector (S3c). Western blot results shown are representative of at least three independent experiments. (B) Colony formation assay showing the colony number of Daoy cells with control vector or S3c after Sant‐1 treatment. Data are presented as mean ± SD; *t*‐test was used to determine the significance. Results shown are representative of at least three independent experiments. (C) WST‐1 assay of relative cell viability showing SMB21‐Gli2ΔN cells were resistant to LDE225 treatment alone (10 µm, 72 h) compared with the LDE225‐treated control cells (SMB21). However, WP1066 treatment (2 µm, 72 h) significantly sensitizes SMB21‐Gli2ΔN resistant cells to LDE225 treatment. Data are presented as mean ± SEM (*n* = 5), *t*‐tests were used to determine the significance.

Treatment targeting Shh signaling induces p21 expression that results in cell cycle arrest and apoptosis [43, 44], while downregulation of p21 leads to drug treatment resistance in cancers [45, 46]. To assess STAT3's role in Shh‐targeted treatment resistance, we generated stable STAT3 knockdown MB cells (STAT3 shRNA) and examined p21 expression. As shown in Fig. 4A (left panel), Daoy and D556 cells from single colonies with STAT3 knockdown expressed higher levels of p21 than shRNA control cells. To confirm that STAT3 regulates p21, we treated Shh MB cells stably transfected with control vector or p‐IRES‐S3c (constitutively activated STAT3, referred to as S3c) [47], with Sant‐1. Sant‐1 treatment markedly induced p21 expression in the control transfected cells, but was completely abrogated in cells with constitutively activated STAT3 (Fig. 4A, right panel), suggesting that STAT3 may play a role in Shh‐targeted drug resistance. To verify whether STAT3 activity mediates drug resistance, we performed colony formation assays with our stable transfected MB cells (control vector or with S3c) treated with vehicle (DMSO) or Sant‐1 for 24 h, then removed the drug treatment, and let the cells continue to grow until fixed and stained for colony count. The average number of tumor colonies formed was not significantly different between DMSO control treatment of vector control cells and the cells with constitutively activated STAT3, 214, and 209, respectively. However, in the Sant‐1‐treated groups, the vector control cells showed an average of only 0.3 colonies after treatment, with the majority of culture dishes having no colonies. In contrast, Sant‐1‐treated cells with constitutively activated STAT3 had up to 4 tumor colonies per culture dish, with an average number of 3.3 colonies (11‐fold higher than control, *P* < 0.05, Fig. 4B). To confirm that STAT3 activity promotes Shh‐targeted drug resistance, we used Smo antagonist LDE225 to treat LDE225‐resistant SMB21‐Gli2ΔN Shh MB cells [13]. LDE225 treatment significantly decreased cell viability by nearly 30% in the control SMB21 cells, relative to the vehicle control treated SMB21 cells, *P* < 0.01. LDE225 treatment had no inhibitory effect on SMB21‐Gli2ΔN cells, confirming the treatment resistance of the SMB21‐Gli2ΔN cells [13]. However, LDE225 treatment of SMB21‐Gli2ΔN cells in the presence of the clinical STAT3 inhibitor WP1066 [48, 49] significantly reduced viability by 35% relative to vehicle control (*P* < 0.01) and significantly lower viability compared with LDE225 or WP1066 treatment alone, (*P* < 0.01, *P* < 0.05, respectively) (Fig. 4C).

### 3.5 STAT3 activity is critical for expression of Hck in Shh MB, whereas STAT3‐driven Gli1 expression requires Smo activation

**Fig. 5:**
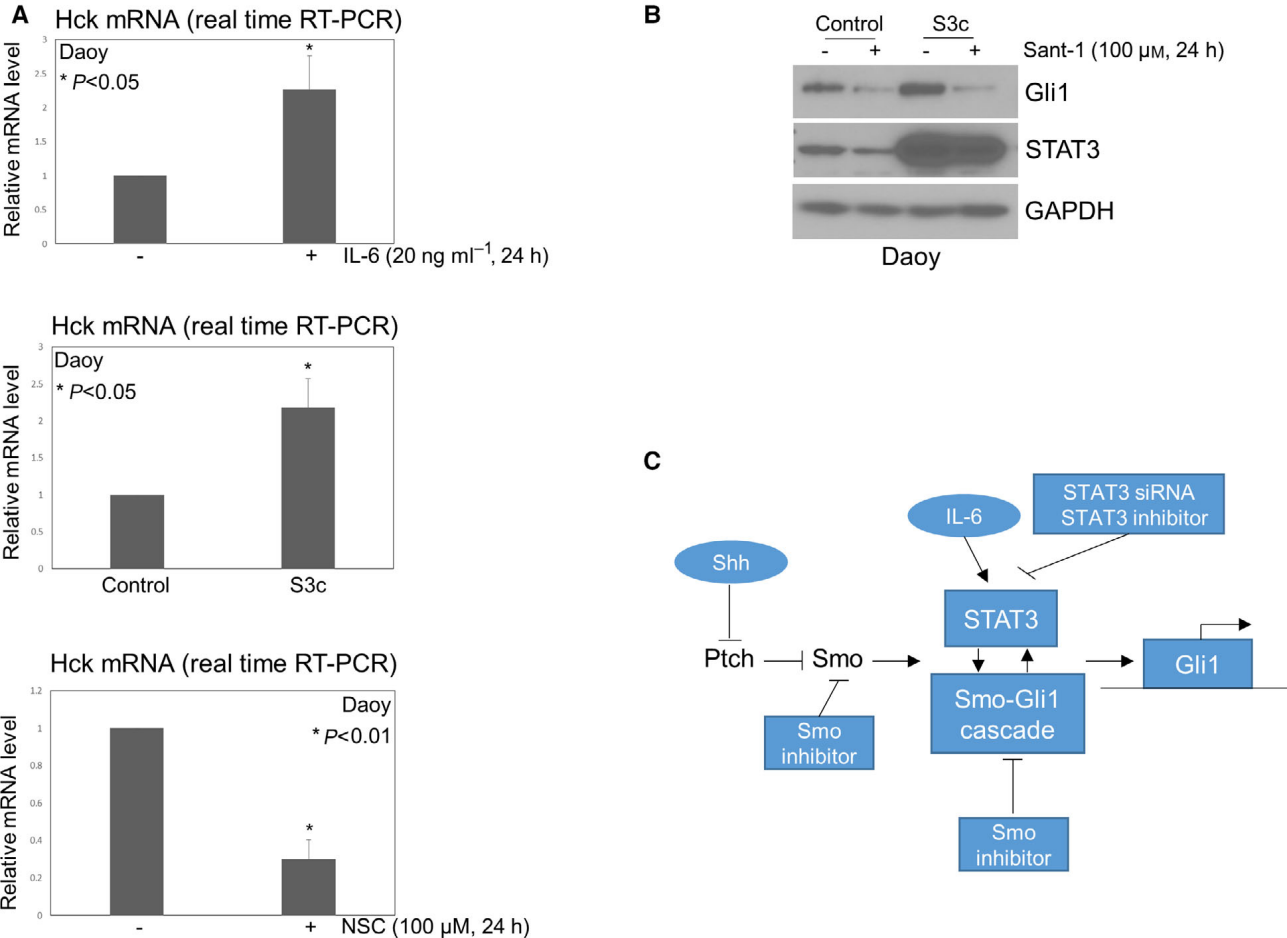
STAT3 activity is critical for expression of Hck in Shh MB, whereas STAT3‐driven Gli1 expression requires Smo activation. (A) Bar graphs of relative mRNA levels by RT‐PCR showing IL‐6 or constitutively activated STAT3 significantly promoted expression of *Hck* in Daoy cells (top and middle panels); NSC74859 treatment significantly decreased expression of *Hck* (bottom panel). Data are presented as mean ± SD; *t*‐tests were used to determine the significance. Results shown are representative of at least three independent experiments. (B) Sant‐1 treatment inhibited expression of Gli1 in Daoy cells with control vector or S3c. Results shown are representative of at least three independent experiments. (C) Schema depicting the interdependent regulation of STAT3 and Shh signaling.

Hck, a Src family member, is highly expressed in Shh MB, and is a Gli1 target gene, forming a positive feedback loop with Gli1 to amplify Shh signaling in MB [50]. Based on our results (Figs 1 and 2), a positive feedback loop also exists for STAT3 and Shh signaling. We thus hypothesized that STAT3 may interact with Hck, and plays an important role in regulation of Hck expression in Shh MB. To address this question, we examined whether STAT3 activity is critical for Hck expression in Shh MB cells. As shown in Fig. 5A (top panel), IL‐6 treatment significantly increased *Hck* expression, 2.3‐fold higher than control treated cells (*P* < 0.05). To confirm that STAT3 promotes *Hck* expression, we examined *Hck* in our constitutively activated STAT3 (S3c) cells. In S3c cells, *Hck* expression is 2.2‐fold higher than in vector control cells (Fig. 5A, middle panel. *P* < 0.05), similar to that of IL‐6 treated control cells. In contrast, STAT3 inhibition by NSC74859 treatment significantly decreased *Hck* expression (Fig. 5A, bottom panel. *P* < 0.01). These data indicate that STAT3 activity is critical for expression of Hck in Shh MB. To elucidate whether STAT3‐driven Gli1 expression requires Smo activation, we treated the vector control and S3c cells with DMSO control or the Smo antagonist Sant‐1, then examined the expression of STAT3 and Gli1. As shown in Fig. 5B, Sant‐1 treatment greatly inhibited STAT3 expression in the vector control cells, but had only slight effect on STAT3 expression in S3c cells, consistent with the effect observed of Sant‐1 treatment (Fig. 4A, right panel). However, Gli1 expression was markedly inhibited by Sant‐1 in both control and S3c cells, indicating that STAT3‐driven Gli1 expression requires Smo activation, STAT3 alone is not sufficient to drive Gli1 expression, while targeted inhibition of any component of STAT3 and Shh signaling is sufficient to suppress Shh signaling (Fig. 5C).

### 3.6 STAT3 activity is critical for Shh‐driven medulloblastoma formation *in vivo*

**Fig. 6:**
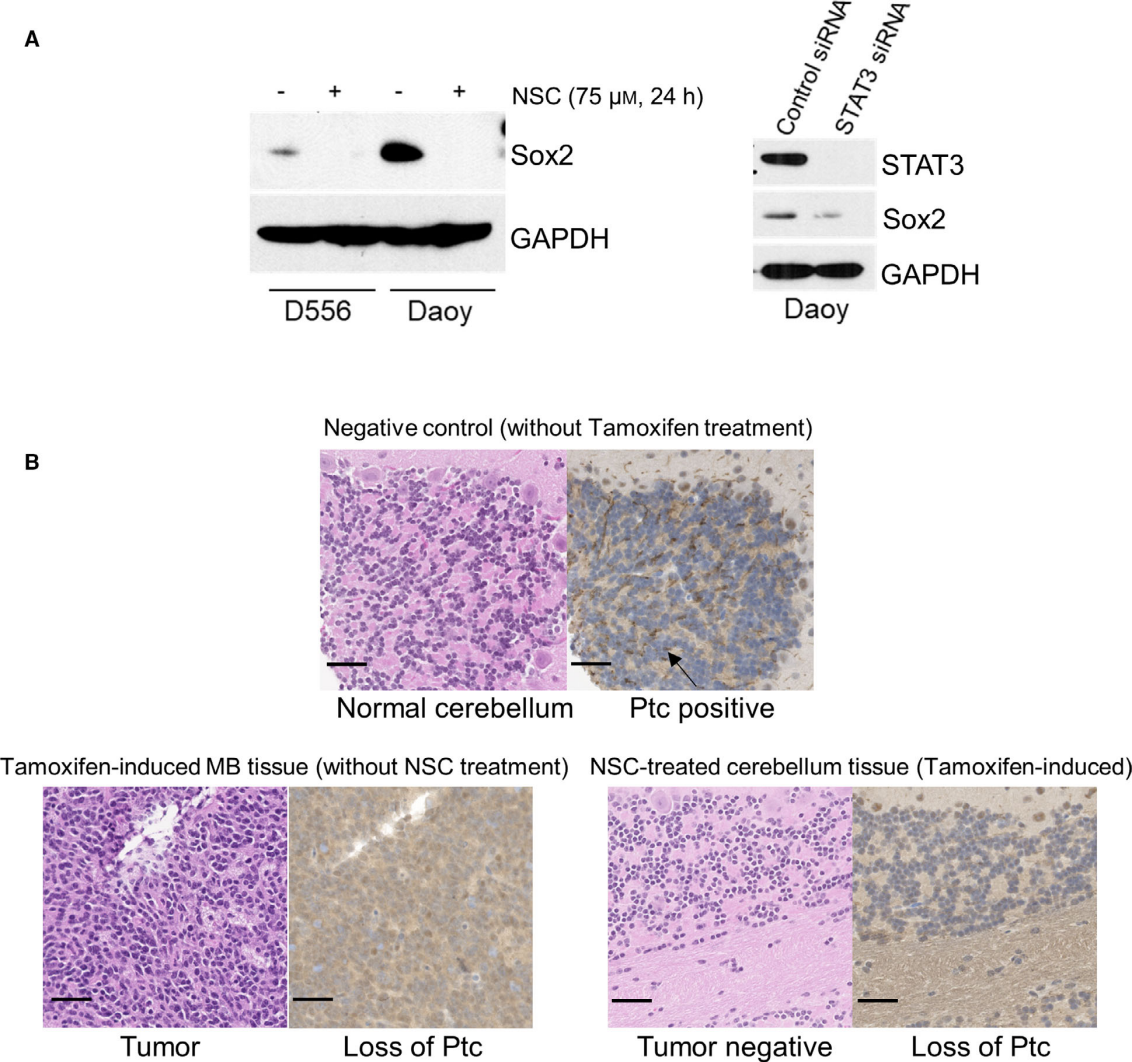
STAT3 activity is critical for Shh‐driven medulloblastoma formation *in vivo*. (A) Western blot of Sox2. Left panel: NSC74859 treatment abolished expression of Sox2 in both D556 and Daoy cells; right panel: STAT3 siRNA‐mediated knockdown of STAT3 led to decrease of Sox2 expression in Daoy cells. Western blot results shown are representative of at least three independent experiments. (B) Representative tissue histopathology from the Math1‐Cre‐ER‐*Ptc* floxed murine model of Shh MB. Left panels: H&E staining of normal cerebellum from Math1‐Cre‐ER‐*Ptc* floxed mouse without tumor (no tamoxifen induction) (top), cerebellar tumor after tamoxifen induction (without NSC74859 treatment) (bottom, left), and NSC74859‐treated tamoxifen‐induced mouse without cerebellar tumor (bottom right panels, left); right panels: immunohistochemistry for Ptc in cerebellar and tumor tissue of corresponding mice (brown staining, arrow in upper right panel points to examples of diffuse positive cell membrane expression in the external granular layer of normal cerebellum). Pictures shown are representative images; scale bars represent 50 µm.

The importance of STAT3 in the maintenance of a wide variety of cancer stem cells is well documented [51, 52, 53]. Sox2 is a transcription factor essential for self‐renewal of neural and the cancer stem cell population reported to drive relapse in Shh MB [54]. To determine whether STAT3 regulates Sox2, we treated cells with NSC74859 or used STAT3 siRNA to inhibit STAT3 activity, then examined Sox2 expression. We observed remarkably decreased Sox2 with either treatment (Fig. 6A), indicating that STAT3 maintains this marker of cancer stem cells.

Based on our data demonstrating a role for STAT3 in regulating the Sox2 expression marker for the cancer stem cell population of Shh MB, we performed an *in vivo* study to determine whether inhibition of STAT3, prior to established tumor development, affects initiation and formation of Shh MB. To address this question, we induced constitutive Shh signaling in the neuron precursor cells of the cerebellum in Math1‐Cre‐ER‐*Ptc* ^flox/flox^ mice by administering tamoxifen to pregnant females after E17.0. In this model, induction of tumor takes place between p1 and p12 and 100% mice have Shh MB by 8–12 weeks [32, 55]. At postnatal day 7, prior to tumor formation by histological examination, pups were randomly assigned to receive the STAT3 inhibitor NSC74859 (50 mg·kg^−1^) or vehicle control by I.P., every other day for 2 weeks, with 20 mice examined for each group (NSC74859‐treated or control treated). Mice were observed daily for signs of toxicity or tumor burden until death/euthanasia or termination of the experiment at 40 weeks, at which time brains were dissected and examined by histopathology for the presence of tumor. There were no signs of treatment‐related toxicity (e.g., depressed activity level or weight loss) in either group, nor any early observable differences in the health between the groups. However, twice as many mice required euthanasia due to neurological signs of excessive tumor burden at an early age (< 14 weeks after birth) in the control group, 10 out of 20 (50%) versus the NSC74859‐treated group, 5 out of 20 (25%). This suggests that inhibition of STAT3 may slow down early growth of initiated MB. At termination of the experiment at 40 weeks, all mice were genotyped and confirmed to have the appropriate Math1‐Cre‐ER‐*Ptc* ^flox/flox^ construct for tumor formation. Notably, 20 out of 20 (100%) mice in the control group developed MB, whereas only 13 out of 20 (65%) mice in the NSC74859‐treated group developed MB (*P* < 0.01). To verify that tamoxifen had sufficiently induced *Ptc* deletion in the NSC74859‐treated group, such that MB should have formed, we performed RT‐PCR of the cerebellar tissue to detect loss of *Ptc* relative to that of tumor tissue derived from control treated mice and normal cerebellum from control Math1‐Cre‐ER‐*Ptc* ^flox/flox^ mice not administered tamoxifen (noninduced). We confirmed that all NSC74859‐treated mice without evidence of tumor by histopathology had appropriate loss of *Ptc*, comparable to control treated tumor‐bearing mice and thus should have formed MB well before 40 weeks (Fig. S3). To confirm *Ptc* loss, we also performed immunohistochemistry for Ptc and showed that the tumor tissue from control treated mice and cerebellar tissue from NSC74859‐treated mice without tumor had similar loss of Ptc expression, relative to the negative control (mouse normal cerebellum tissue without tamoxifen treatment) (Fig. 6B). Of tumors that formed in the NSC74859‐treated cohort, no differences were observed in tumor morphology, infiltration, survival, or growth as evidenced by tumor size at time of dissection compared with control treated tumors. Since the genetic mouse model has 100% tumor formation, unless inhibition occurs between p1‐p12 of mouse the tumors will form by 8–12 weeks. Thus, the significant reduction of tumor formation after STAT3 inhibitor treatment indicates the essential role of STAT3 in Shh MB development.

## 4 Discussion

Shh MB is dependent on constitutive Shh pathway signaling for tumor initiation and growth [2, 4]. Targeting Shh signaling is thus an attractive therapeutic strategy against Shh MB. Clinical investigations of Smo antagonists showed very promising early results, but ultimately failed due to intrinsic and acquired drug resistance, concluding that multiple pathway targeting may be necessary for effective long‐term disease control [11, 12, 14]. Recently, tumor‐associated astrocyte secretion of Shh was shown to be required for Shh MB tumorigenesis, despite the absence of the Shh receptor Ptch1, indicating that a novel Ptch1‐independent Shh pathway is involved in MB progression [31]. Activation of the transcription factor STAT3 has been reported in association with aberrant Shh signaling in carcinogenesis [26, 27], while evidence indicates elevated STAT3 protein expression and phosphorylation in Shh MB and that STAT3 mediates transcription of Shh target genes [56]. Furthermore, low STAT3 expression has been shown to correlate with improved survival in Shh MB patients [56]. Herein, we demonstrate a novel critical interdependent relationship between STAT3 and Smo, forming a positive feedback loop to promote STAT3 activity and Smo‐mediated signaling and target gene transcription, which in turn maintains Shh MB survival, proliferation and treatment resistance to Smo antagonists *in vitro*, and Shh‐driven MB formation *in vivo*.

A similar critical interdependency between STAT3 and Smo‐driven oncogenesis has been reported in SmoM2 murine skin tumors [27]. In that study, the addition of a Smo agonist also induced STAT3 activation, while removal of STAT3 from the mouse epidermis or disruption of IL‐11Ra/STAT3 signaling significantly reduced SmoM2‐driven tumor development. In comparison, we show that not only STAT3 activation is Smo‐dependent, but also expression of STAT3, similarly induced by treatment of Shh MB cells with either Shh or IL‐6, a primary activator of STAT3 [57, 58], is Smo‐dependent. Likewise, Shh or IL‐6 treatment alone significantly induces Gli1, and these effects are STAT3‐dependent. In liver cancer stem cells, Shh/Gli‐regulated cell functions were also found to be mediated by activation of the IL‐6/STAT3 pathway, but whether there is a similar direct effect of Shh‐blockade on IL‐6‐mediated STAT3 signaling was not reported [59]. Of interest is that in addition to Shh, astrocytes and microglia secrete IL‐6 [60, 61], and it was reported that treatment with excretory/secretory products (ESP) of fifth‐stage larval *A. cantonensis* induces IL‐6 secretion and activates Shh signaling in mouse astrocytes, and ESP‐induced IL‐6 is dependent on Shh signaling [62]. Thus, IL‐6 derived from astrocytes may also contribute to Shh MB tumorigenesis. It remains unknown whether STAT3 activity in Shh MB cells by non‐IL‐6 activators, such as IL‐10 and IL‐11, is similarly co‐dependent on Smo activity. Importantly, similar to that observed in SmoM2 murine skin tumors, blockade of STAT3 in our mouse model resulted in significant disruption of Smo‐driven MB tumor induction. While blockade of tumor formation did not occur in all mice, it is plausible that tumor initiation in our study may have temporally preceded drug administration and/or drug penetration to the stem‐like cell population was not complete in a portion of mice tested. This is supported by our finding no differences in the histopathology, tumor growth rate or survival in the portion of mice that still formed tumors after treatment in the first week of life compared with controls.

Mechanistically, we observed that the Src kinase family member Hck functions in a positive feedback loop with Gli1 to amplify Shh signaling in MB, as has been reported [50]. Hck activation can be triggered by IL‐6 in MYD88‐mutated Waldenström macroglobulinemia and diffuse large B‐cell lymphoma [63]. In addition, co‐expression of Hck and STAT3 in 293T cells potently activates STAT3 [64], but an interaction between STAT3 and Hck has not been reported in MB. Herein, we demonstrated that IL‐6, or constitutively activated STAT3, significantly promotes *Hck* expression, and that maintenance of *Hck* basal level depends on STAT3. Since we showed that Smo‐mediated Shh signaling is reciprocally dependent on STAT3, and STAT3 activity is required for Hck expression in Shh MB, our data support a wider interactions among STAT3, Hck, and Smo that mediates Shh signaling amplification that is critically co‐dependent on Smo/STAT3. Moreover, we found that Smo inhibition decreases Gli1 in cells with constitutively activated STAT3, indicating that STAT3 alone is not sufficient to maintain Gli1 expression. Indeed, we observed that independent loss of function of STAT3 or Smo will similarly result in loss of Gli1 expression.

In addition to intrinsic and acquired mutations, several reports have indicated alternative pathway activation as a mechanism of resistance to Smo antagonist treatment of Shh MB [11, 12, 65]. For example, RAS/MAPK, and PI3K pathway activation have been shown to drive resistance to Smo inhibition [13, 66]. Notably, IL‐6 promotes the activation of STAT3, RAS/MAPK and PI3K pathways, and crosstalk between STAT3 and RAS/MAPK with PI3K has been implicated in carcinogenesis and treatment resistance [67, 68, 69]. In our study, we found that STAT3 regulates Sox2 expression, a critical neural stem cell factor observed in association with the treatment‐resistant cell population of relapsed Shh MB [54]. Further, we observed that STAT3 suppresses both basal level and Smo antagonist‐induced p21 expression, which plays an important role in mediating drug resistance in response to anticancer treatment. In concordance with these findings, we show that constitutive STAT3 activation mediates resistance to Smo inhibitor treatment of Smo‐activated Shh MB cells, while dual blockade of STAT3 and Smo with the respective clinical inhibitors WP1066 and LDE225 effectively kills LDE225‐resistant Gli2‐activated Shh MB cells. Likewise, combination treatment with Smo and STAT3 inhibitors resulted in synergistic killing of Shh MB cells. Since our data indicate a co‐dependency of the cells on Smo and STAT3, combined inhibition is likely necessary to overcome Smo inhibitor‐resistant cells. In this case, we speculate that STAT3 inhibition alone is sufficient to block the Gli2‐mediated effects, possibly by disrupting Gli2‐DNA binding, but upstream functional Smo still needs to be inhibited to block all of Smo effector mechanisms. The precise dependency and interactions of the Smo‐STAT3 axis in Shh MB remain to be elucidated. Further investigation is also needed to determine whether STAT3‐mediated treatment resistance is through the activation of RAS/MAPK and PI3K/AKT pathways or other mechanisms. We attempted investigation of combined Smo and STAT3 inhibitor treatment *in vivo*; however, due to the potency of Smo inhibitors alone in our murine model, resulting in tumor eradication for many months, the model is not adequate for investigating synergy or Smo inhibitor resistance *in vivo*. In addition, our *in vivo* study focuses only on the essential role of STAT3 in tumor initiation and early tumor formation, not the impact of STAT3 inhibition on established tumor growth, in which mouse weight, tumor size, mouse survival, and STAT3 expression and other tumor histopathological features would all be relevant analyses. Current studies are underway to examine the effect of STAT3 inhibition on established Shh MB tumor growth and mouse survival to support clinical investigation of STAT3 inhibitors against medulloblastoma.

## 5 Conclusions

We demonstrate in Shh MB that STAT3 is required for Shh signaling and also demonstrate that STAT3 activity is critical for expression of Hck in Shh MB cells. Further, combined treatment with Smo and STAT3 inhibitors results in potent synergistic killing of Shh MB cells and overcomes Smo antagonist treatment resistance of Gli2‐driven Shh MB cells. Importantly, treatment with a STAT3 inhibitor significantly blocks tumor formation in a murine transgenic model of Shh‐driven MB. Thus, targeting STAT3 is a potential novel therapy to treat Shh MB and potentially overcome Smo antagonist drug resistance.

## Conflict of interest

The authors declare no conflict of interest.

## Author contributions

LY participated in the design of the study, generated stable cell lines expressing control vector or constitutively activated STAT3; conducted western blots, Gli1 reporter assays, siRNA transfections, cell proliferation and viability assays, and colony formation assays; performed data analysis; and drafted the manuscript. HZ generated stable cell lines expressing control shRNA or STAT3 shRNA and conducted real‐time RT‐PCR and western blots. JL performed western blot, real‐time RT‐PCR, *in vivo* study, and immunohistochemistry. AM and AD performed immunostaining for p‐STAT3. BY performed flow cytometry to analyze cell apoptosis. KKJ reviewed manuscript and provided valuable comments on revision of the manuscript. LFM prepared and grew primary mouse Shh MB cells and conducted western blot. MJS conducted histopathological review for mouse brain tissues. TJM directed all experiments and provided the oversight for all data analysis, result interpretation, and the draft of the final manuscript. All the authors have read and approved the final manuscript.

### Peer Review

The peer review history for this article is available at https://publons.com/publon/10.1002/1878‐0261.13097.

## Supporting information


**Fig. S1.** Effects of STAT3 and Smo inhibitors on expression of p‐STAT3 in MB cells.

**Fig. S2.** STAT3 inhibitor treatment inhibits p‐STAT3 and induces decrease of cell viability in Shh MB cells.

**Fig. S3.** Expression of *Ptc* mRNA in cerebellar tissues from mouse model of Shh MB.

## Data Availability

The supporting data for this study are contained within the manuscript as [Link], [Link], [Link].
