# Supplementary figures and images for: STAT3 is required for Smo‐dependent signaling and mediates Smo‐targeted treatment resistance and tumorigenesis in Shh medulloblastoma
Source: Mol Oncol. 2021 Sep 25;16(4):1009–25. doi: 10.1002/1878-0261.13097 (PMC8847987; doi:10.1002/1878-0261.13097)

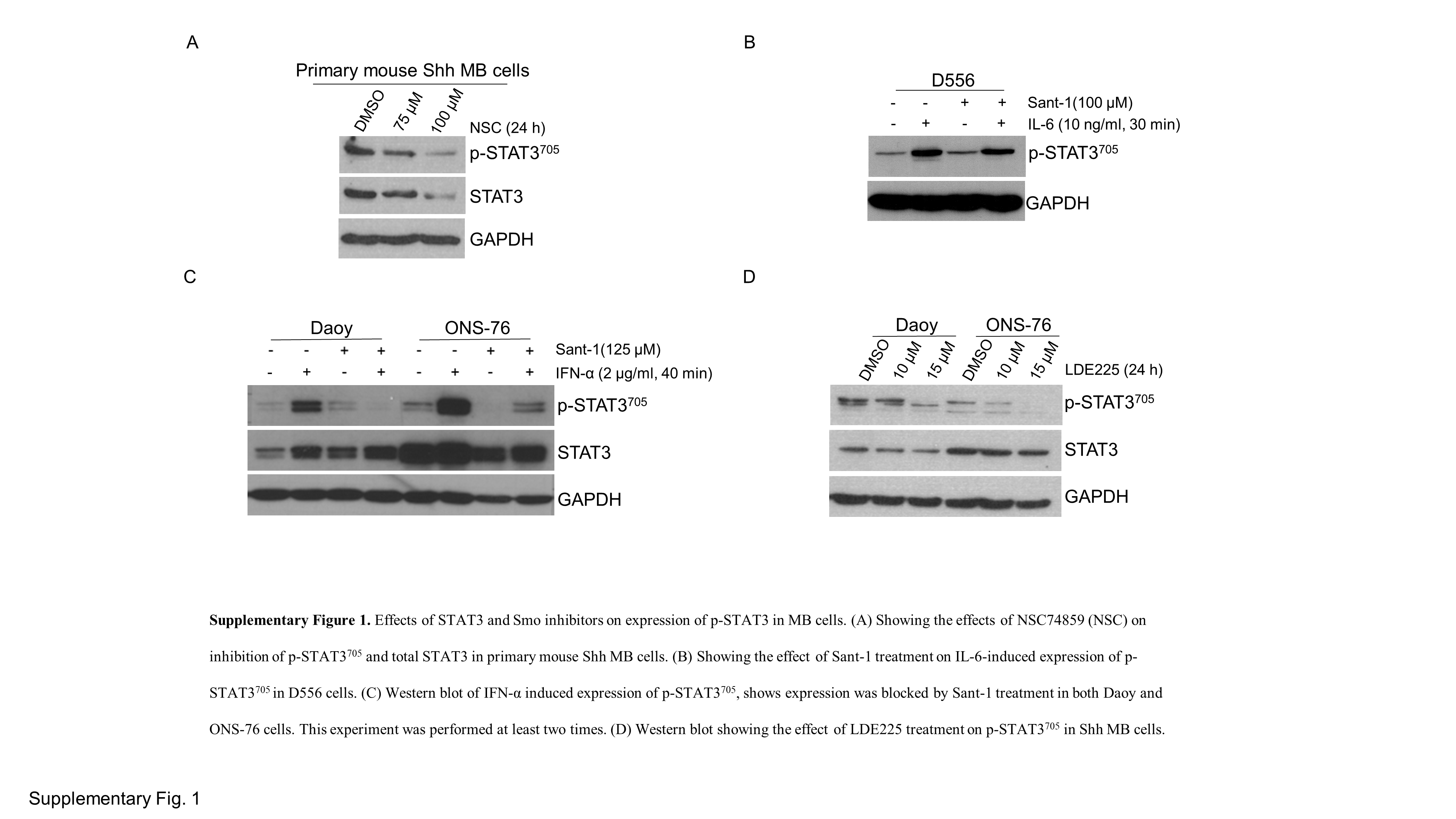

Supplement: Supplementary file 1 — Fig. S1. Effects of STAT3 and Smo inhibitors on expression of p‐STAT3 in MB cells. [file MOL2-16-1009-s001.tif]

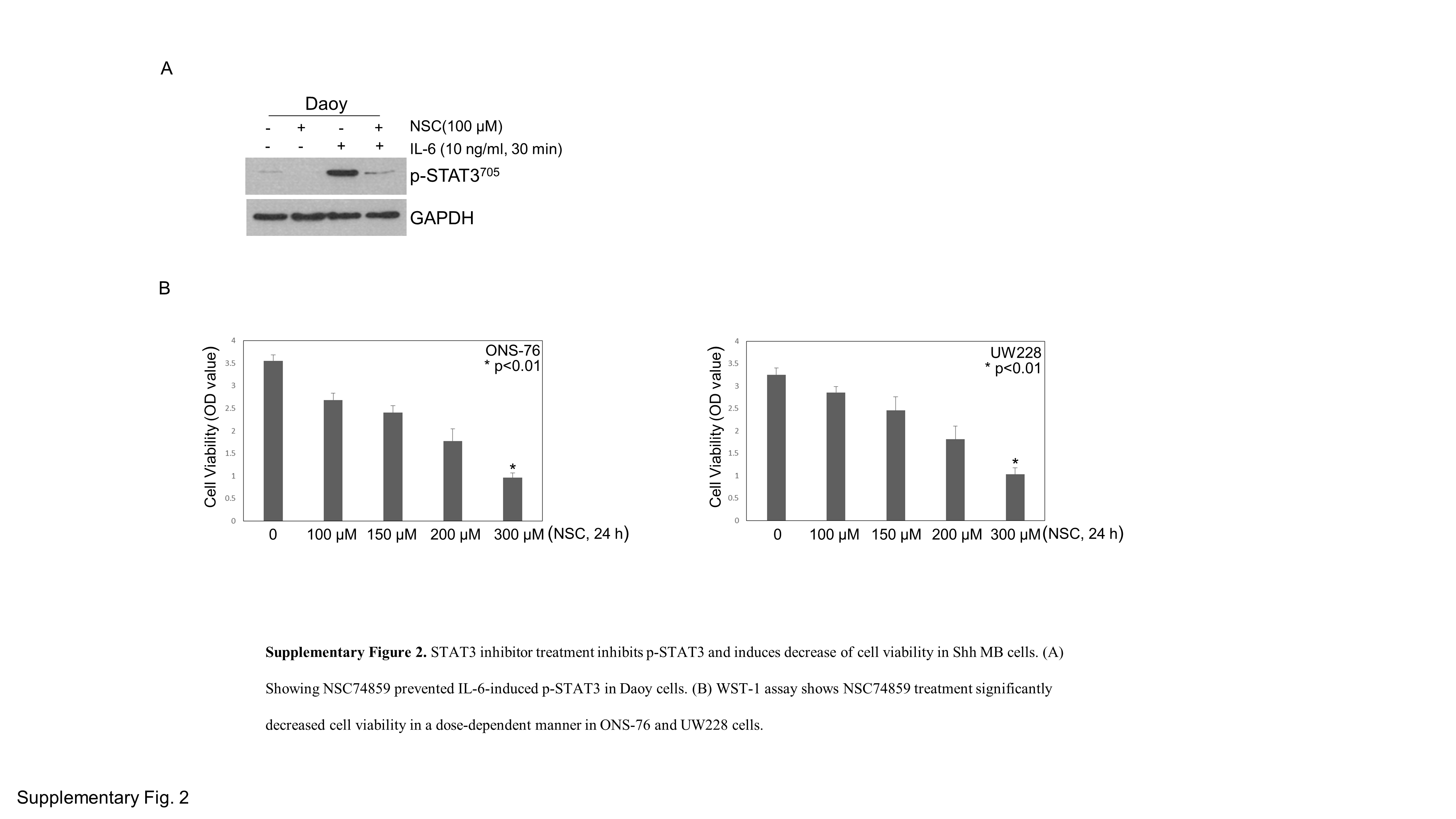

Supplement: Supplementary file 2 — Fig. S2. STAT3 inhibitor treatment inhibits p‐STAT3 and induces decrease of cell viability in Shh MB cells. [file MOL2-16-1009-s002.tif]

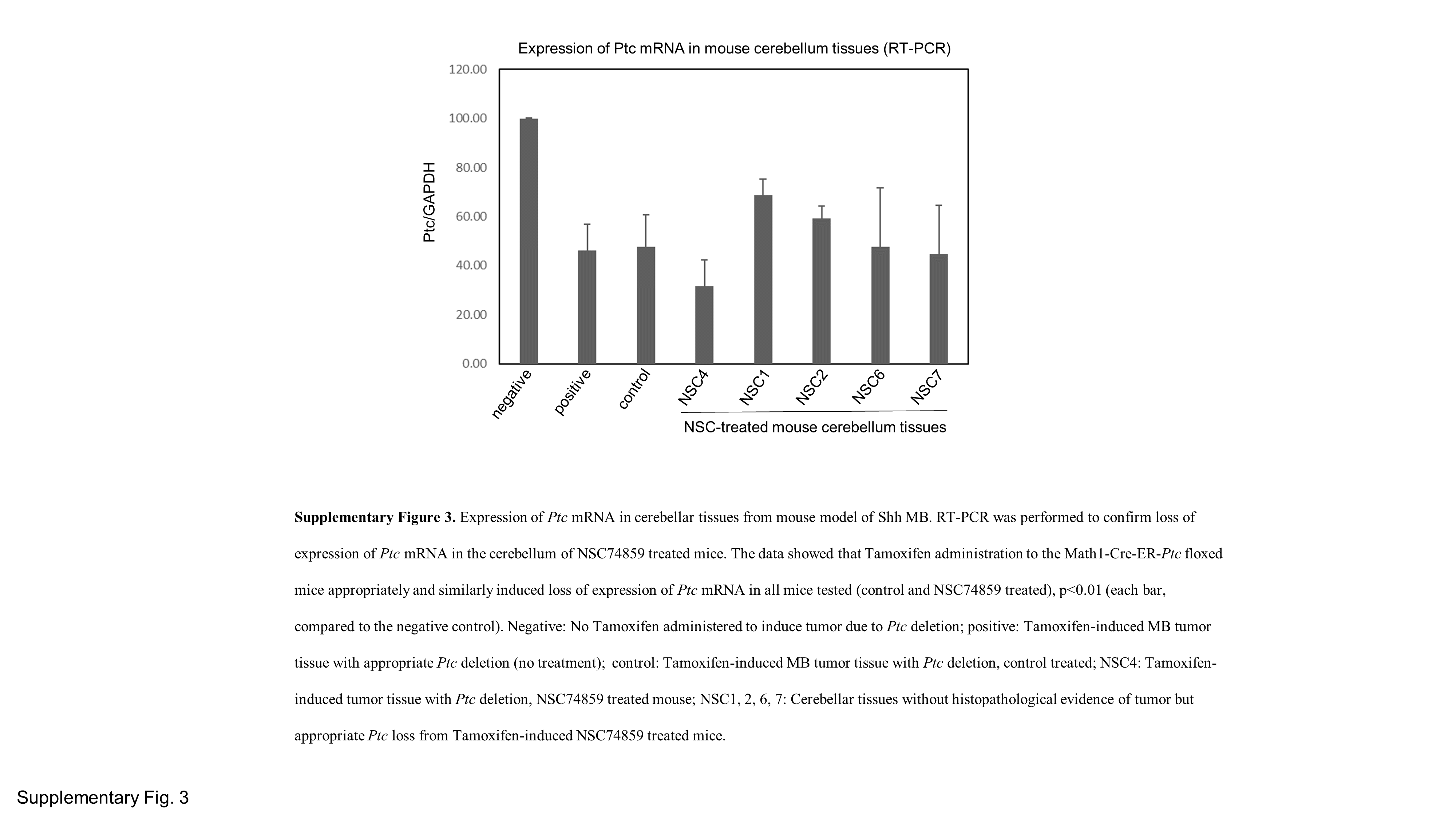

Supplement: Supplementary file 3 — Fig. S3. Expression of Ptc mRNA in cerebellar tissues from mouse model of Shh MB. [file MOL2-16-1009-s003.tif]
